# Impact of a Serious Game on the Intention to Change Infection Prevention and Control Practices in Nursing Homes During the COVID-19 Pandemic: Protocol for a Web-Based Randomized Controlled Trial

**DOI:** 10.2196/25595

**Published:** 2020-12-15

**Authors:** Laurent Suppan, Mohamed Abbas, Gaud Catho, Loric Stuby, Simon Regard, Stephan Harbarth, Sophia Achab, Mélanie Suppan

**Affiliations:** 1 Division of Emergency Medicine Department of Anesthesiology, Clinical Pharmacology, Intensive Care and Emergency Medicine University of Geneva Hospitals and Faculty of Medicine Geneva Switzerland; 2 Infection Control Programme WHO Collaborating Centre on Patient Safety University of Geneva Hospitals and Faculty of Medicine Geneva Switzerland; 3 Genève TEAM Ambulances Geneva Switzerland; 4 Division of General Surgeon Geneva Directorate of Health Geneva Switzerland; 5 Specialized Facility in Behavioral Addictions ReConnecte Geneva University Hospitals Geneva Switzerland; 6 WHO Collaborating Center in Training and Research in Mental Health University of Geneva Geneva Switzerland; 7 Division of Anesthesiology Department of Anesthesiology, Clinical Pharmacology, Intensive Care and Emergency Medicine University of Geneva Hospitals and Faculty of Medicine Geneva Switzerland

**Keywords:** COVID-19, transmission, serious game, infection prevention, health care worker, SARS-COV-2, nursing home, randomized controlled trial, elderly, older adult, infection control, infectious disease

## Abstract

**Background:**

Nursing home residents are at high risk of complications and death due to COVID-19. Lack of resources, both human and material, amplifies the likelihood of contamination in these facilities where a single employee can contaminate dozens of residents and colleagues. Improving the dissemination of and adhesion to infection prevention and control (IPC) guidelines is therefore essential. Serious games have been shown to be effective in developing knowledge and in increasing engagement, and could motivate nursing home employees to change their IPC practices.

**Objective:**

Our aim is to assess the impact of “Escape COVID-19,” a serious game designed to enhance knowledge and application of IPC procedures, on the intention of nursing home employees to change their IPC practices.

**Methods:**

We will carry out a web-based randomized controlled trial following the CONSORT-EHEALTH (Consolidated Standards of Reporting Trials of Electronic and Mobile Health Applications and Online Telehealth) guidelines and incorporating relevant elements of CHERRIES (Checklist for Reporting Results of Internet E-Surveys). Participants will be randomized to either the control or the serious game (intervention) group. First, both groups will be asked to answer a questionnaire designed to gather demographic data and assess baseline knowledge. The control group will then receive a quick reminder of the current national guidelines and links to IPC guidelines for health care professionals, while the other group will play the game. Both groups will then have to answer a second questionnaire designed to assess their willingness to change their IPC practices after having followed their respective material. After completing this questionnaire, they will be granted access to the material presented to the group they were not assigned to and receive a course completion certificate. The primary outcome will be the proportion of participants willing to change their IPC practices according to group. Secondary outcomes will include the analysis of specific questions detailing the exact changes considered by the participants. Factors associated with participant willingness or reluctance to change behavior will also be assessed. Attrition will also be assessed at each stage of the study.

**Results:**

The study protocol has been presented to our regional ethics committee (Req-2020-01262), which issued a declaration of no objection as such projects do not fall within the scope of the Swiss federal law on human research. Data collection began on November 5, 2020, and should be completed by December 4, 2020.

**Conclusions:**

This study should determine whether “Escape COVID-19,” a serious game designed to improve compliance with COVID-19 safe practices, modifies the intention to follow IPC guidelines among nursing home employees.

**International Registered Report Identifier (IRRID):**

DERR1-10.2196/25595

## Introduction

### Background and Importance

Nursing home residents are at high risk of complications and death if they develop symptoms of COVID-19 after being infected with SARS-CoV-2 [1-4]. If infected, a single nursing home employee can potentially contaminate dozens of residents and colleagues [5], since allowing the virus to enter nursing homes leads to rapid interresident transmission [6]. Many long-term care facilities (LTCFs) were ill-prepared to face the first wave of the pandemic and should be helped as much as possible to prevent health care–associated transmission during potential future waves [7]. Such waves seem all the more likely as new COVID-19 cases have been identified since September 2020 in LTCFs located in Geneva, Switzerland, 14 weeks after the last infection was diagnosed here [8]. In addition to aiding efforts to combat the pandemic and to avoid infection, showing a high level of support for nursing home employees may also enhance their motivation [9]. Indeed, since the start of the pandemic, many researchers have pointed out the dramatic lack of resources, both human and material, faced by many LCTFs [10-13].

In nursing homes as in other facilities, viral transmission is often facilitated by the suboptimal application of infection prevention and control (IPC) guidelines [14]. Accordingly, a recent systematic review has identified the promotion of hand and respiratory hygiene and the use of appropriate personal protective equipment to be some of the most critical IPC practices that could help prevent viral transmission among nursing home residents and staff [15]. Dissemination of these guidelines and practices might however be hampered by the current need for physical and social distance [16]. Moreover, application of IPC guidelines may be jeopardized by the presence of divergent and sometimes contradicting messages [17,18], and even by mistrust in guidelines issued by health care authorities [19].

The probability of actually executing an action is strongly linked to the intention of performing it [20]. By increasing engagement and developing knowledge [21,22], serious games could prove instrumental regarding the effective dissemination of IPC guidelines and the promotion of COVID-19 safe practices [23,24]. Using Nicholson’s [25] concept of meaningful gamification, we recently developed “Escape COVID-19,” a serious game specifically designed to motivate health care workers in adopting good IPC practices [26]. Indeed, building and strengthening their intrinsic motivation might be at least as important as reminding them of the most current guidelines to help avoid infection [27]. The usefulness and cost-effectiveness of computer-based serious games is however still debated, and previous studies have pointed out a considerable lack of evidence regarding this education modality [28].

### Objective

Our principal objective is to assess the impact of this serious game on the intention of nursing home personnel to change their IPC practices. We will also aim to determine the factors explaining the reasons that motivate change and those explaining the lack of willingness to change one’s behavior.

## Methods

### Study Design and Setting

**Figure 1:**
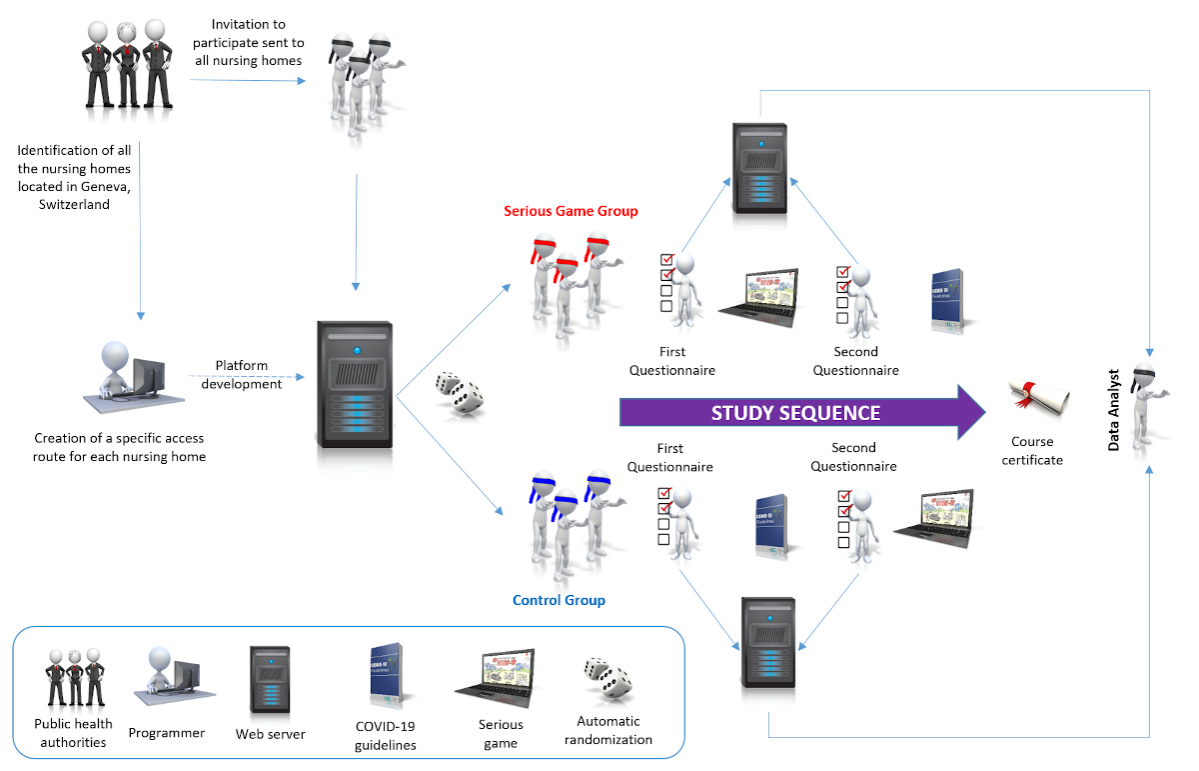
Study design and sequence.

We will carry out a web-based, triple-blind (investigator, participants, and data analyst) randomized controlled trial, following the CONSORT-EHEALTH (Consolidated Standards of Reporting Trials of Electronic and Mobile Health Applications and Online Telehealth) guidelines [29]. Elements from CHERRIES (Checklist for Reporting Results of Internet E-Surveys) will be included when relevant [30]. The design and sequence are summarized in Figure 1.

A list of all the nursing homes located in Geneva was obtained through the city’s public health authorities. All staff from these nursing homes will be invited to take part in this study, on a voluntary basis, regardless of their professional status. Information regarding the study and its objectives, including data security, will be provided. Consent will be gathered electronically.

### Online Platform

A specific and fully automated platform created using the latest version of the Joomla content management system [31] and hosted on a Swiss server will be used in this study. All data will be stored on an encrypted, MySQL-compatible database. Only one author (LSu) will be able to access the database. The platform will be secured by the RSFirewall (RSJoomla) [32] and Admin Tools (Akeeba) [33] components. The questionnaires will be administered using Community Surveys Pro (Corejoomla) [34], which allows for the use of branching logic and for the export of responses in CSV format. The Membership Pro (Joomdonation) component will be used to handle registrations [35]. Randomization will be achieved by the GegaByte Random Article module (GegaByte Technologies) [36]. Therefore, randomization will be fully automated, and participants or investigators will not be able to influence group allocation.

Access to the different steps of the study sequence will be managed through Joomla’s native access control list (PHP functions *JUserHelper::addUserToGroup* and *JUserHelper::removeUserFromGroup*). PHP functions will be embedded using Sourcerer (Regular Labs) [37]. Redirect-on-Login (Pages-and-Items) will be used to allow users to immediately access the appropriate section when resuming their study path [38]. Certificates will be generated using RSForm!Pro (RSJoomla) [39]. Daily backups will be scheduled using a cron job script and uploaded on a physically separate server through an encrypted connection.

### First Questionnaire

Participants will be asked to fill in 2 questionnaires. Immediately after activating their account, the first questionnaire will be displayed. This questionnaire is designed to gather demographic data and to assess the initial level of knowledge regarding SARS-CoV-2 transmission and IPC guidelines. To limit attrition, the number of initial questions will be kept at a minimum and branching logic will be used to avoid displaying irrelevant items. The structure of this questionnaire, the original questions in French and their translation in English, are displayed in Multimedia Appendix 1.

### Serious Game

The experiment will be conducted using version 2.1.1 of the “Escape COVID-19” serious game [26], which is freely available on the internet [40]. This serious game has been created under Storyline 3 (Articulate Global) and can be played on many different platforms, including smartphones and tablets, due to its HTML5 compatibility. The game was designed using the SERES framework [41] and Nicholson’s RECIPE (reflection, engagement, choice, information, play, exposition) for meaningful gamification [25]. It is made of 4 different levels representing the typical phases that most health care employees experience daily. To make the game more engaging, the graphics included in the game were designed by Eric Buche, a well-known Swiss cartoonist [42].

**Figure 2:**
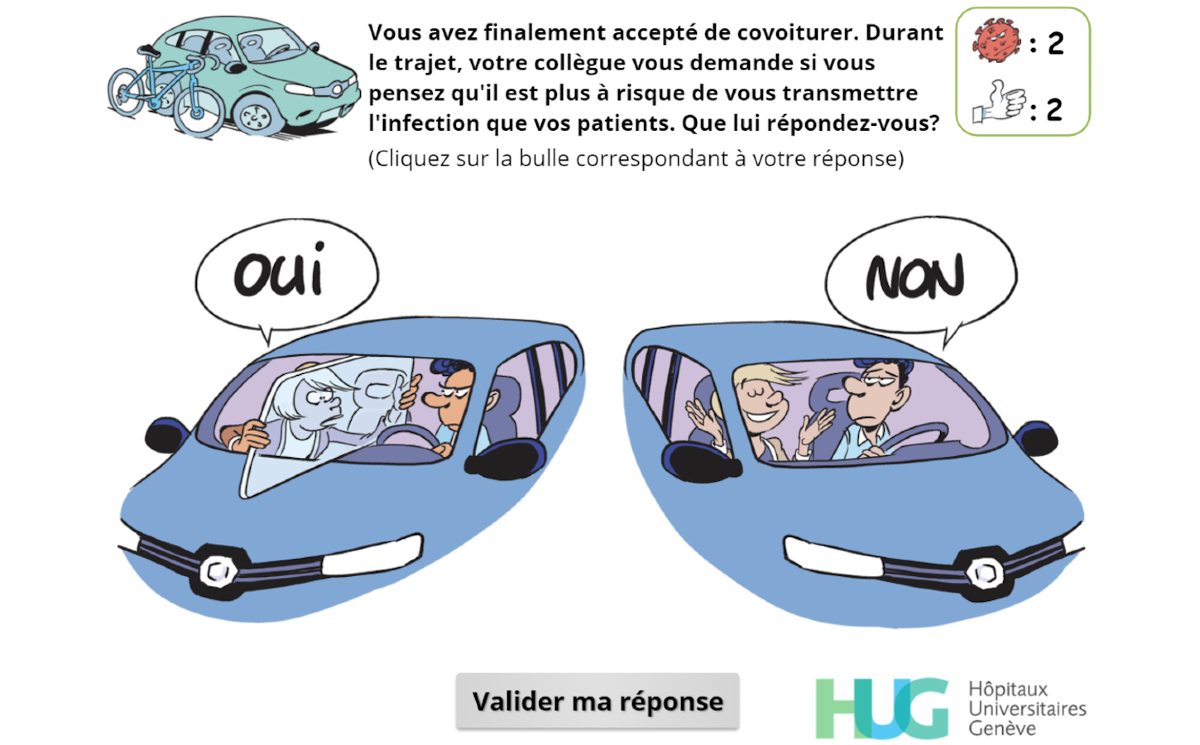
Simple-choice interaction.

**Figure 3:**
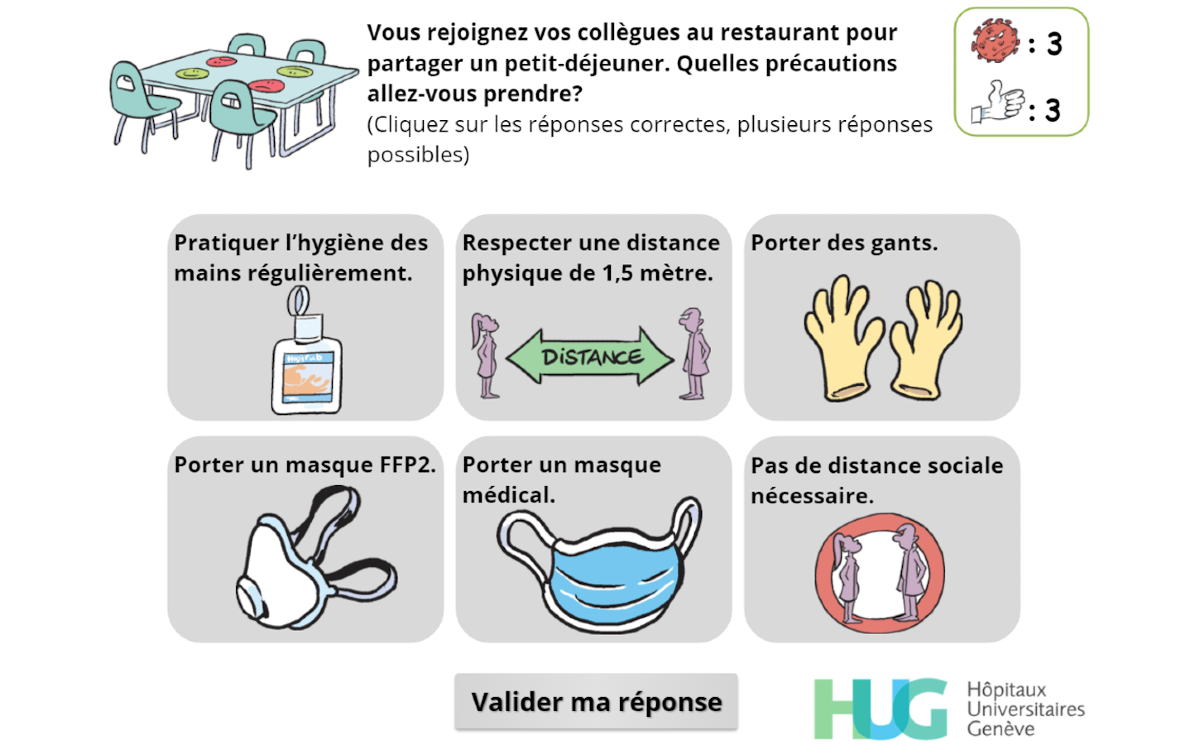
The player has to identify the precautions they should take when joining their colleagues for breakfast.

Throughout the game, players are asked to make choices (Figure 2) or to answer questions directly related to the exposition element (Figure 3), which aims at creating a meaningful narrative in the serious game [25].

**Figure 4:**
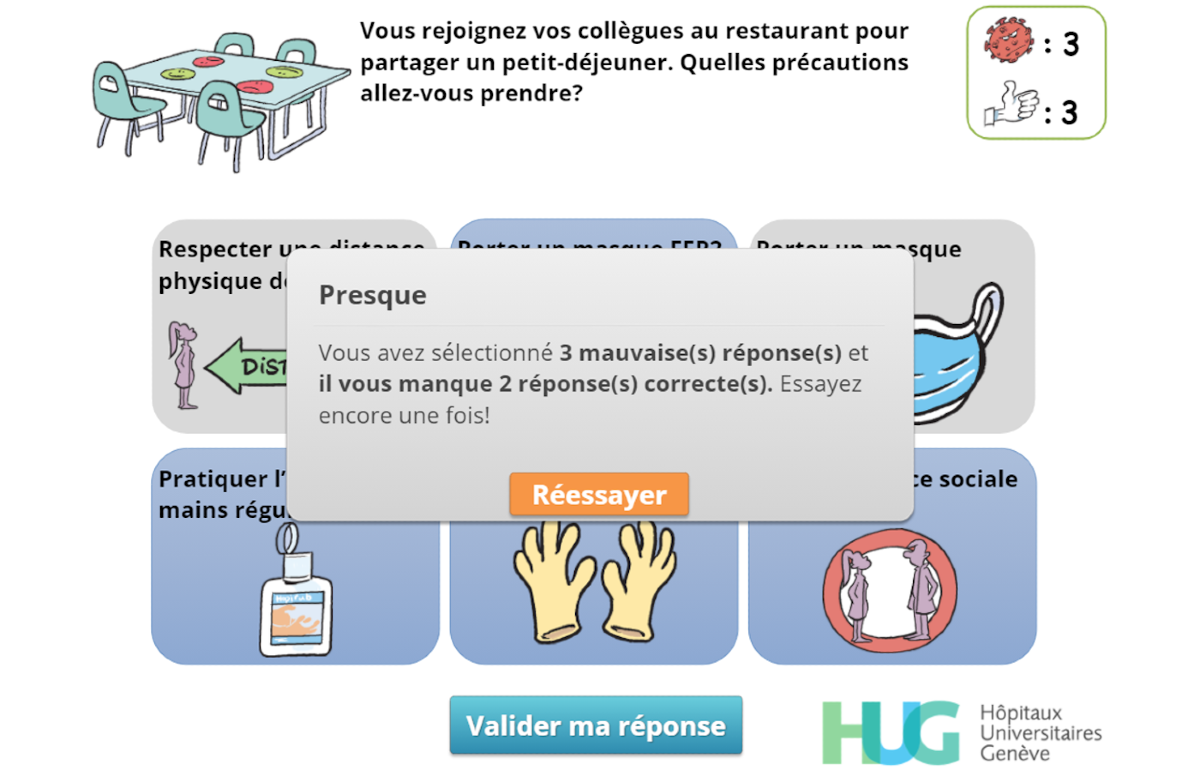
Feedback. The player has submitted an initial answer and is informed that they have selected 3 wrong answers (“3 mauvaises réponses”) and that 2 correct answers are missing (“il vous manque 2 réponses correctes”). They can retry (“Réessayer”) once.

**Figure 5:**
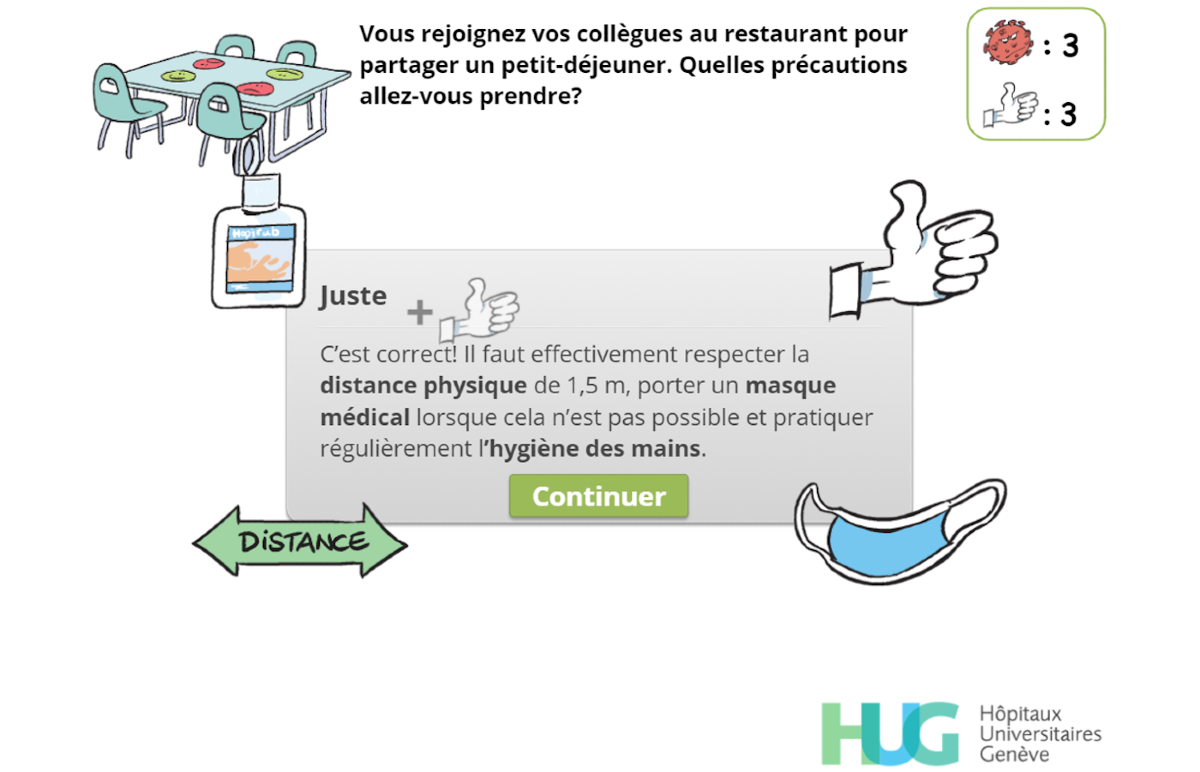
Feedback. The user has correctly answered the question. Visual hints related to the correct answers are displayed (mask, distance arrow, alcohol-based handrub) and a short text emphasizes the expected answers. A thumbs-up image and a plus sign appear, rise, and progressively fade out before the thumbs-up count is updated.

**Figure 6:**
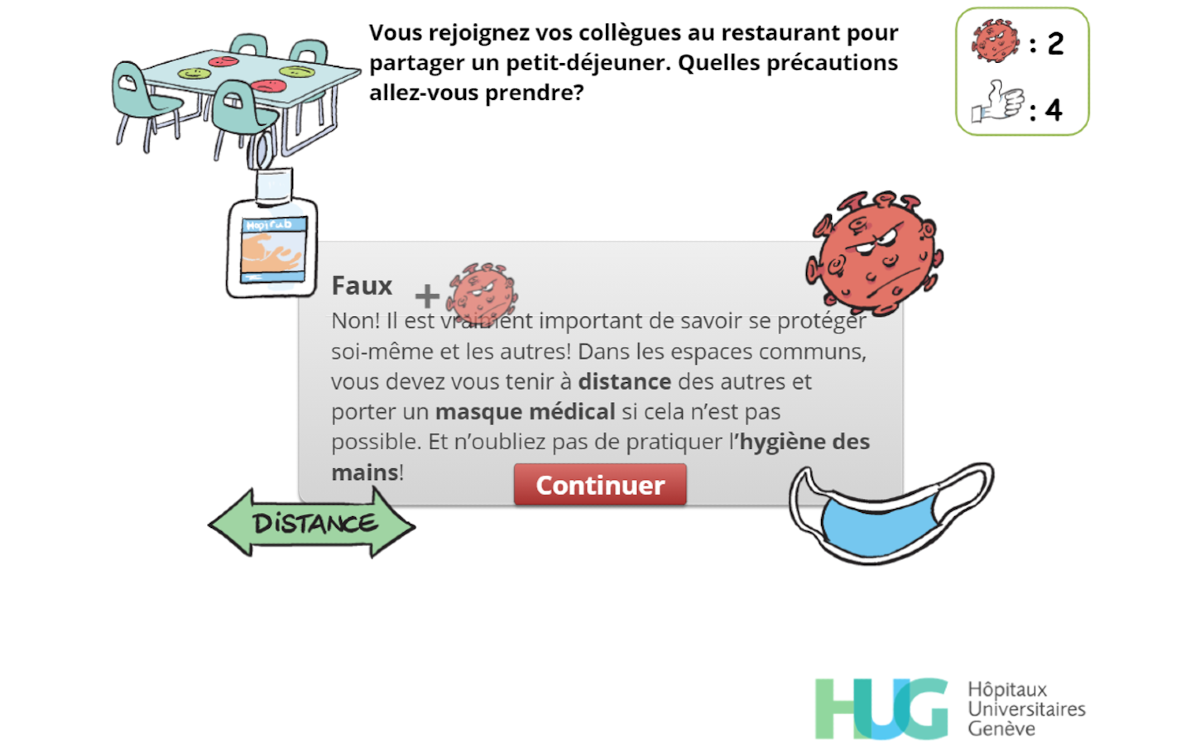
Feedback. The player has retried but has failed to identify the correct answers. Visual hints related to the correct answers are displayed (mask, distance arrow, alcohol-based handrub) and a short text emphasizes the expected answers, which will be displayed when the player clicks on continue (“Continuer”). A virus and a plus sign appear, rise, and progressively fade out before the virus count is updated.

**Figure 7:**
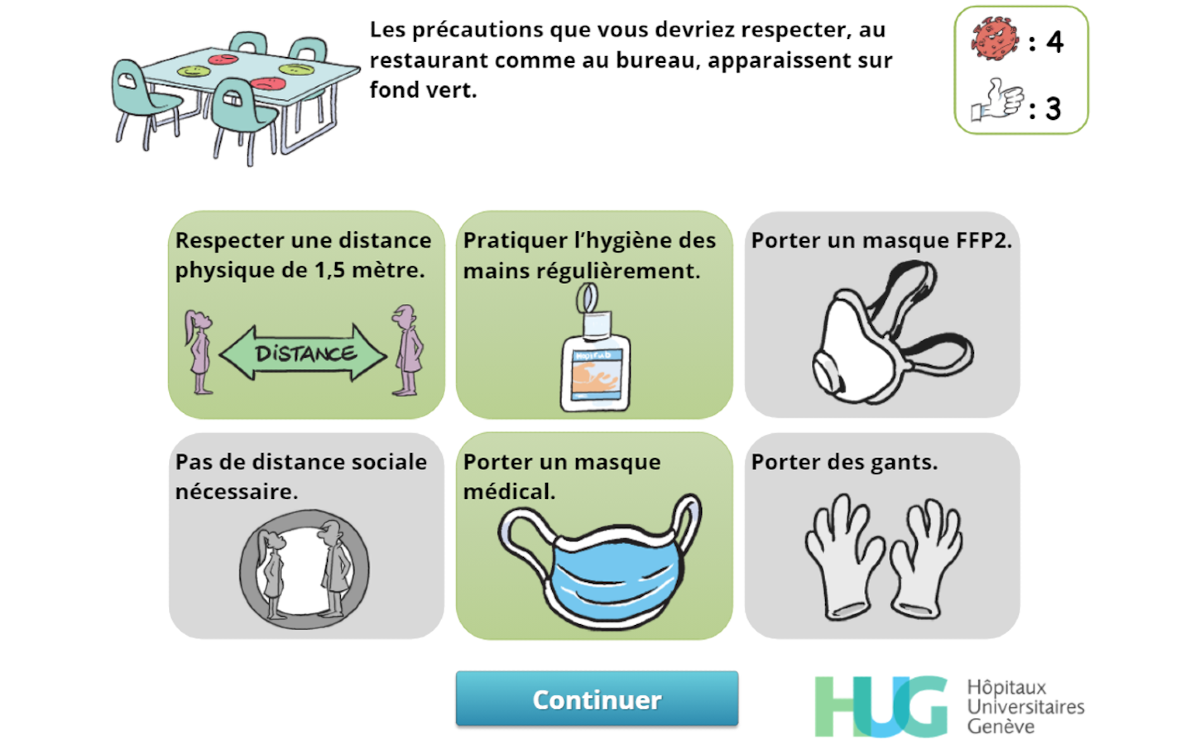
Feedback. When the user has failed to correctly answer the question, the correct answers are displayed with a green background.

Feedback is used extensively [43] to allow the player to correct an answer (Figure 4) and to reinforce the expected behavior (Figures 5-7).

**Figure 8:**
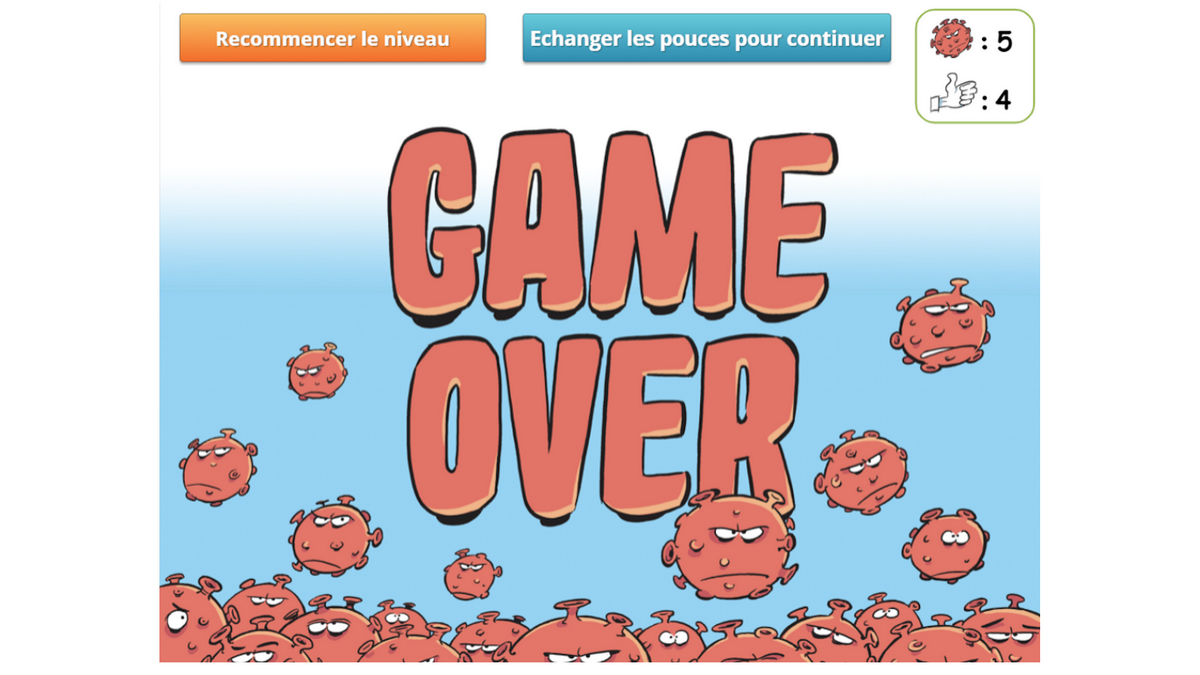
Game-over screen. The player has accumulated 5 viruses and can either spend their thumbs up to decrease the virus count or restart the level.

Each time the player performs a desirable action or selects the correct answer, a “thumbs up” is awarded. Conversely, the player gets a red virus for each incorrect answer or behavior. If the player accumulates a total of 5 viruses, a game-over screen is displayed (Figure 8). The player can then choose to spend their thumbs up to decrease the virus count (1:1) or to restart the level.

### Control Materials

The control materials include a quick reminder of the current national guidelines published by the Federal Office of Public Health of the Swiss Confederation [44] and links to IPC guidelines for health care professionals (Vigigerme) provided by the Geneva University Hospitals and freely available on the internet [45].

### Second Questionnaire

The second questionnaire is designed to assess whether the participants intend to change their IPC practices after completing the first set of learning materials (Multimedia Appendix 2). Therefore, the control group will complete it after seeing the standard guidelines, and the serious game group after finishing the game. Once again, branching logic will be used to try to limit attrition.

All multiple-choice and multiple-answer questions are mandatory. A completeness check will be performed at the end of each survey page. Mandatory questions will be highlighted and will have to be answered before allowing participants to move on to the next step. There will be no way to change the answers after completing a page.

### Outcomes

The primary outcome will be the proportion of nursing home employees reporting they are willing to change their IPC practices according to group.

Secondary outcomes will be a composite of the questions based on a 5-point or 6-point Likert scale according to group. Each individual question will also be assessed. We will also aim at identifying the factors associated with participant willingness to change their behavior and analyze the reasons given by participants who are opposed to making changes [46].

We will also assess attrition at each stage of the study according to group [47-50].

### Participants and Sample Size

Health authorities were asked for a comprehensive list of the email addresses of all nursing home employees working in Geneva, to construct a convenience sample. We decided to include all employees, regardless of their professional status or of the potential specificities of the facilities they work in, without any exclusion criteria. To detect a difference of 10% at the .05 significance level with a power of 80%, 388 participants will be needed for each group. The number of eligible employees is estimated to be approximately 4000 people. Therefore, a participation rate of around 20% will be required.

All employees will be invited to participate regardless of their professional status. Participation will be encouraged by delivering a course completion certificate upon completing the study path. No financial incentives will be provided.

To avoid potential duplicates, participants will be required to register on the site using a valid email address. No other personal information, including names, will be asked for during the registration process. The system will automatically send an activation email to check whether the email address provided is valid. Participants will be told that clicking the activation link will be considered as consent to participate in the study.

### Statistical Analysis

Stata 15.1 (StataCorp LLC) will be used for data curation and statistical analysis. Data will be curated by the first author (LSu) and neutral names will be randomly assigned to the control and serious game groups before transferring the DTA file to the blinded data analyst (LSt). To avoid any potential conflicts of interest, the analyst was not part of the serious game development team and did not participate in the original publication describing its development. There will be no interim analysis.

Incomplete answer sets will be excluded. Imputation techniques will not be used. Answer sets marked as completed should not contain any missing value by virtue of the completeness checks automatically performed by the survey component.

Univariate and multivariable logistic regression will be used to assess the primary outcome. Adjustment will be done according to prior knowledge (expressed as percentage of correct answers), professional status, and nursing home. The expected sample size should prevent overfitting. We will check the log-linearity assumption graphically and test the goodness of fit using the Hosmer-Lemeshow test.

The analysis of secondary outcomes will be carried out by assigning numerical values to the answers gathered through the use of Likert scales. As the 6-point Likert scale ranges from 1 (not at all) to 6 (very much), the same numbers (ie, a score ranging from 1 to 6) will be used for each item. The composite outcome will be the sum of the 9 questions and will be analyzed using univariate and multivariable linear regression analyses, with the same adjustment variables as the primary outcome. Each question will be analyzed separately.

For the 5-point Likert scale, values ranging from –2 to +2 will be assigned to each answer, with positive values attributed to changes enhancing IPC behavior. A composite outcome will be generated, which will be the sum of these values. We made the choice to treat this discrete variable as continuous and use a linear regression analysis, first univariate, then multivariable (with the same adjustment variables as the primary outcome). The same weight will be applied to all questions when computing composite outcomes. As a reduction in the use of N95 respirator masks can also be considered as enhancement depending on the setting, a sensitivity analysis will be done by analyzing the composite outcome with and without the N95 respirator mask item.

Descriptive statistics will be used to detail the factors associated with participant willingness to change or refuse to change behavior. The Student *t* test and the chi-square test will be used to assess differences between groups.

The curated data file will be made available on the Mendeley Data repository.

## Results

The study protocol has been presented to our regional ethics committee (Req-2020-01262), which issued a declaration of no objection as such projects do not fall within the scope of the Swiss federal law on human research [51]. The public health authorities of Geneva did not have access to a list of email addresses of all nursing home employees. However, they provided us with a comprehensive list of all nursing homes to allow us to create specific access routes for each nursing home. They were reluctant to provide us with the email addresses of nursing home managers but were nevertheless willing to send information and invitation emails on our behalf.

The online platform was finalized on November 3, 2020 [52]. It was created by authors LSu and MS and thoroughly tested by all coauthors. We provided the health care authorities with a generic email template (Multimedia Appendix 3) and a list of nursing home–specific accreditations, which acted as passwords to prevent participants from enlisting under the wrong nursing home. This email template informed the recipients that, should they agree to participate, all data would be processed anonymously but could and would be used for research purposes. The email stated that the study path would let participants access IPC guidelines as well as a serious game but did not tell them in which order these materials would be accessed. The approximate time required to complete the whole path (30 minutes) was given, along with an email address that could be used to contact the investigators. Participants were also told that they would receive a course completion certificate after completing the study path.

Data collection began on November 5, 2020, and is scheduled to end on December 4, 2020.

## Discussion

### Main Considerations

This study should help determine whether a serious game can improve the adoption of IPC guidelines among nursing home personnel. This game should appeal to at least 3 of the 4 types of players described by Bartle [53] in 1996: achievers, who might want to gather all the thumbs up while avoiding getting a single virus to get the highest score possible; explorers, who might find the narrative created through the use of the exposition element of Nicholson’s RECIPE appealing; and socializers, who might associate the use of the thumbs up sign with social networks. Nevertheless, some participants might be recalcitrant to this kind of intervention. Identifying the profile of these participants and the reasons underlying their resistance to change could help either improve the game or devise better targeted interventions [46]. Conversely, the identification of factors enhancing the adoption of safe IPC practices will help explore ways of strengthening COVID-19 safe messages.

To avoid a potential conflict of interest as 5 of the authors of this protocol were also members of the team that developed the serious game, the data analyst, who will be blinded, was not part of the development team.

### Limitations

Some limitations can already be anticipated. First, as we were unable to obtain a comprehensive list of all potential participants, and because we cannot be sure that nursing home managers will actually transmit the information to their personnel, we will be prevented from determining the actual number of potential participants. While this could lead us to underestimate the participation rate, another mechanism could result in overestimating this rate. Indeed, we will have no way of preventing nursing home–specific accreditations to be transferred to third parties, and some participants might not be part of the target population. To alleviate this concern, we will, upon request, create specific accreditations to allow other categories of personnel to create accounts on the platform and follow the study path. Any data gathered through the use of such accreditations will not be included in the analysis.

Despite its design, which is intended to attract different types of players, this serious game might be more successful for certain profiles. Because we decided on a limited number of questions to try to reduce attrition, we elected not to include questions pertaining to the identification of the player type. Other studies would therefore be needed to explore a potential correlation.

The convenience sample used in this study might not be representative of other systems. Moreover, even though only 20% of the target population will be required to participate to reach our estimated sample size, we cannot be certain of the participation rate. A low participation rate will intrinsically carry the risk of a selection bias.

Another important limitation is that, even though the theory of planned behavior has proven its worth in the field many times, we will have no way of proving that the intention of adopting COVID-19 safe IPC practices correlates with actual changes in the field. Direct observations should be performed to ascertain this fact, but limitations in human resources and funds will prevent us from carrying such observations as part of the present study.

Finally, the importance of rapidly deploying this study and the serious game did not allow us to wait for the peer-review process to be completed before proceeding with the study. Therefore, we will be unable to change either the study design or the questionnaires despite the valuable input the reviewers will provide us with.

### Conclusion

This study should determine whether “Escape COVID-19,” a serious game designed to improve compliance with COVID-19 safe practices, modifies the intention of applying IPC guidelines in nursing homes.

## Appendix

Multimedia Appendix 1 First questionnaire.

Multimedia Appendix 2 Second questionnaire.

Multimedia Appendix 3 Generic email template.

## Abbreviations

CHERRIES: Checklist for Reporting Results of Internet E-Surveys
CONSORT-EHEALTH: Consolidated Standards of Reporting Trials of Electronic and Mobile Health Applications and Online Telehealth
IPC: infection prevention and control
LTCF: long-term care facility
RECIPE: reflection, engagement, choice, information, play, exposition
