# Supplementary material for: Impact of a Serious Game on the Intention to Change Infection Prevention and Control Practices in Nursing Homes During the COVID-19 Pandemic: Protocol for a Web-Based Randomized Controlled Trial
Source: JMIR Res Protoc. 2020 Dec 15;9(12):e25595. doi: 10.2196/25595 (PMC7744143; doi:10.2196/25595)
Supplement: Multimedia Appendix 1 [file resprot_v9i12e25595_app1.docx]

**Multimedia Appendix 1.** First questionnaire, designed to gather demographic data and assess the baseline knowledge level of nursing home employees.

| Page | Field | Original Question | English Translation |
| --- | --- | --- | --- |
| 1 | Demographics | Vous êtes:   - Un homme - Une femme | You are:   - A man - A woman |
|  |  | Quel est votre âge? | What is your age? |
|  |  | Vous faites principalement partie du personnel:   - Médical - Soignant - Administratif/support - Autre ^a^ | You are mainly part of the:   - Medical staff - Nursing staff - Administrative/support staff - Other ^a^ |
|  |  | Vous êtes:^b^   - Infirmier.e - Assistant.e en soins et santé communautaire - Aide-soignant.e - Physiothérapeute - Autre ^a^ | You are:^b^   - Nurse - Nursing assistant - Health care assistant - Physiotherapist - Other ^a^ |
|  |  | Vous êtes en contact avec des patients:^c^   - Très fréquemment - Assez fréquemment - Peu fréquemment - Quasiment jamais | You are in contact with patients :^c^   - Very frequently - Quite frequently - Seldom - Almost never |
|  |  | Depuis combien d'années travaillez-vous dans le domaine de la santé? | For how many years have you worked in health care? |
| 2^d^ | Baseline knowledge | Vous vous apprêtez à entrer dans la chambre d’un patient COVID-19 **qui n’est pas sous CPAP** pour lui prodiguer des soins, quels équipements de protection sont nécessaires?   - Masque médical - Surblouse - Gants - Tablier de soins - Protection oculaire - Masque ultrafiltrant (FFP2) | You are about to enter a room to care for a COVID-19 patient **who is not receiving CPAP treatment**. What protective equipment should you wear?   - Face mask - Gown - Gloves - Protective apron - Eye protection - N95 mask |
|  |  | Vous vous apprêtez à entrer dans la chambre d’un patient COVID-19 **qui est sous CPAP** pour lui prodiguer des soins, quels équipements de protection sont nécessaires?   - Masque médical - Surblouse - Gants - Tablier de soins - Protection oculaire - Masque ultrafiltrant (FFP2) | You are about to enter a room to care for a COVID-19 patient **who is under CPAP**. What protective equipment should you wear?   - Face mask - Gown - Gloves - Protective apron - Eye protection - N95 mask |
|  |  | Quelle est durée médiane d’incubation du COVID-19 (en jours)?   - 2-3 jours - 4-6 jours - 7-10 jours - 11-14 jours | What is the median incubation time of COVID-19 (in days)?   - 2-3 days - 4-6 days - 7-10 days - 11-14 days |
|  |  | Parmi les situations suivantes, lesquelles doivent conduire à porter un masque ultrafiltrant (FFP2) chez un patient COVID-19 (confirmé ou suspecté)?   - Auscultation d’un patient COVID-19 qui tousse - Traitements par nébulisation - Oxygénation à haut débit - Séance de physiothérapie de rééducation à la marche - Oxygénothérapie nasale à moins de 2L/min | In which of the following situations should you wear an N95 respirator mask when taking care of patient with a COVID-19 infection (confirmed or suspected)?   - Auscultation of a coughing COVID-19 patient - Nebulization therapy - High flow oxygen therapy - Gait rehabilitation physiotherapy session - Nasal oxygen therapy (flow less than 2L/min) |
|  |  | Parmi les suivantes, dans quelles situations le port de protections oculaires est-il recommandé ?   - En cas de contact anticipé avec un liquide biologique par éclaboussure - En cas de contact de proximité avec un patient qui présente de symptômes respiratoires, même en l’absence de diagnostic de COVID-19 - En tout temps dans la chambre d’un patient COVID-19 - En tout temps dans toute situation dans tous les lieux de l’établissement de soins | In which of the following situations should you wear eye protection?   - If contact with a biological liquid (splashing) is anticipated - In case of close contact with a patient with respiratory symptoms, even without a diagnosis of COVID-19 - Anytime when in the room of a COVID-19 patient - At all times in any situation in all areas of the care facility |
| 2^e^ | Baseline knowledge | Dans les lieux communs de l’institution, quels équipements de protection doivent être portés lorsque la distance de 1.5 m ne peut pas être respectée?   - Le masque médical - Les gants - Le masque ultrafiltrant (FFP2) - Les protections oculaires - La surblouse | In the common areas of the institution, what protective equipment should be worn when the distance of 1.5 m cannot be respected?   - A face mask - Gloves - An N95 respirator - Eye protection - A protective gown |
|  |  | Parmi les suivantes, dans quelles situations le port de protections oculaires est-il recommandé ?   - En cas de contact anticipé avec un liquide biologique par éclaboussure - En cas de contact de proximité avec un patient qui présente de symptômes respiratoires, même en l’absence de diagnostic de COVID-19 - En tout temps dans la chambre d’un patient COVID-19   En tout temps dans toute situation dans tous les lieux de l’établissement de soins | In which of the following situations should you wear eye protection?   - If contact with a biological liquid (splashing) is anticipated - In case of close contact with a patient with respiratory symptoms, even without a diagnosis of COVID-19 - Anytime when in the room of a COVID-19 patient   At all times in any situation in all areas of the care facility |
|  |  | Si vous vous trouvez dans la même pièce qu’un patient atteint de COVID-19 qui est sous CPAP, vous devez porter un masque ultrafiltrant (FFP2)   - Vrai - Faux | If you are in a room with a COVID-19 patient who is under CPAP, you must wear an N95 respirator mask   - True - False |
|  |  | Le bio-nettoyage standard de la chambre d’un patient COVID-19 nécessite l’utilisation d’un désinfectant standard (Des-sur®)   - Vrai - Faux | Standard bio-cleaning of a COVID-19 patient's room requires the use of a standard disinfectant (Des-sur®)   - True - False |
| 3 | Symptoms and screening | Si j’ai des symptômes compatibles avec le COVID-19 :   - J’attends de voir l’évolution et en cas de persistance des symptômes je vais me faire tester - Je vais me faire tester le jour même - Si les symptômes sont légers, j’attends que les symptômes passent et je continue de travailler sans faire de test | If I have symptoms compatible with COVID-19:   - I monitor their evolution and get tested if the symptoms persist - I get tested right away (on the same day) - If the symptoms are mild, I wait for the symptoms to recede and continue to work without getting tested |
|  |  | Parmi les symptômes suivants, quels sont ceux qui devraient vous conduire à faire un test de dépistage du COVID-19?   - Rhume - Eruption cutanée - Toux - Maux de gorge - Perte de l'odorat - Maux de tête | Which of the following symptoms should prompt you to get tested for COVID-19?   - Symptoms compatible with a "cold" - Skin rash - Cough - Sore throat - Loss of smell - Headache |

^a^Answering “other” allows the participant to enter free text in a specific field.

^b^Displayed only to participants who identify as part of the “nursing staff.”

^c^Displayed only to participants who identify as part of the “administrative/support staff” or as “other.”

^d^These questions are only displayed to members of the medical or nursing staff.

^e^These questions are only displayed to participants who identify as part of the “administrative/support staff” or as “other.”
