# Supplementary material for: Impact of a Serious Game on the Intention to Change Infection Prevention and Control Practices in Nursing Homes During the COVID-19 Pandemic: Protocol for a Web-Based Randomized Controlled Trial
Source: JMIR Res Protoc. 2020 Dec 15;9(12):e25595. doi: 10.2196/25595 (PMC7744143; doi:10.2196/25595)
Supplement: Multimedia Appendix 2 [file resprot_v9i12e25595_app2.docx]

**Multimedia Appendix 2.** Second questionnaire, designed to determine the intention of changing prevention infection and control practices in nursing home employees.

| Original Question | English Translation |
| --- | --- |
| Après avoir vu ce matériel de formation/information, allez-vous modifier certaines de vos pratiques de prévention de l'infection?   - Oui - Non | After seeing this training / information material, are you going to change any of your infection prevention practices?   - Yes - No |
| Quels domaines ces changements vont-ils concerner?^a,b^   - Le fait de ne pas aller au travail si vous présentez des symptômes compatibles avec le COVID-19 - Le fait de vous protéger autant de vos collègues que de vos patients - La séquence d'habillage lors de procédures AVEC risque d'aérosolisation - La séquence d'habillage lors de procédures SANS risque d'aérosolisation - Le fait de changer plus fréquemment de gants non stériles - Le fait de vous désinfecter les mains plus fréquemment - Le fait de désinfecter votre place de travail - Le fait de manipuler le masque médical avec plus de précautions - Le fait de vous protéger également des personnes asymptomatiques | What areas will these changes affect? ^a,b^   - Not going to work if you have symptoms compatible with COVID-19 - Protecting yourself from both your colleagues and your patients - The donning sequence when dealing with procedures CARRYING a risk of aerosolization - The donning sequence when dealing with procedures NOT CARRYING a risk of aerosolization - Changing non-sterile gloves more frequently - Practicing hand hygiene more frequently - Disinfecting your workplace - Handling the face mask more carefully - Protecting yourself from asymptomatic people as well as from symptomatic ones |
| Vous allez désormais employer: ^a,c^   - Les masques médicaux - Les masques FFP-2 - Les protections oculaires - Les gants non stériles | You are now going to use: ^a,c^   - Face masks - N95 respirator masks - Eye protections - Non-sterile gloves |
| Qu'est ce qui a grandement participé à votre intention de modifier vos pratiques? ^a^   - L'information contenue dans le matériel de formation - Le sentiment de jouer un rôle important dans l'effort commun contre l'épidémie - La probabilité de contaminer un proche - Il faut suivre les procédures - Autre ^d^ | Which of these elements greatly contributed to your intention to modify your practices? ^a^   - The information given in the training material - The feeling of playing an important role in the common effort against the epidemic - The probability of infecting a relative - One should follow the procedures - Other ^d^ |
| Pour quelles raisons vos pratiques ne changeront-elles pas? ^e^   - Le matériel que je viens de consulter était inadapté à ma situation - J'applique déjà toutes les mesures proposées - Le matériel que je viens de consulter n'était pas utile - Je ne crois pas que ces mesures soient utiles - Je suis en désaccord avec les mesures proposées ^d^ - Autre ^d^ | Why will your practices not change? ^e^   - This material was not in line with my situation - I already apply all these guidelines - The material I have just seen was not helpful - I do not believe these measures to be useful - I disagree with these measures ^d^ - Other ^d^ |
| Qu'est-ce qui aurait pu favoriser la modification de vos pratiques?   - Mieux comprendre les raisons justifiant les recommandations - Une probabilité plus importante de contaminer un proche - Le sentiment que vous avez un role important dans l'effort commun contre l'épidémie - Autre ^d^ - Rien - aucun argument ne pouvait me convaincre | What could have motivated you to change your practices?   - Better understand the reasons behind the recommendations - A greater probability of infecting a relative - The feeling of having an important role in the common effort against the epidemic - Other ^d^ - Nothing – I could not have been convinced by any argument |

^a^Question displayed only to participants who answered they were going to change their practices.

^b^Answers based on a 6-point Likert scale (from 1, “not at all,” to 6, “very much”).

^c^Answers based on a 5-point Likert scale (from 1, “much less,” to 5, “much more”).

^d^Ticking this option allows the participant to enter free text in a dedicated field.

^e^Question displayed only to participants who answered they were not going to change their practices.
