# Supplementary material for: Impact of a Serious Game on the Intention to Change Infection Prevention and Control Practices in Nursing Homes During the COVID-19 Pandemic: Protocol for a Web-Based Randomized Controlled Trial
Source: JMIR Res Protoc. 2020 Dec 15;9(12):e25595. doi: 10.2196/25595 (PMC7744143; doi:10.2196/25595)
Supplement: Multimedia Appendix 3 [file resprot_v9i12e25595_app3.pdf]

Bonjour,

Voici les informations de connexion à « Echappe au COVID-19 », un parcours de formation pour parfaire les gestes qui permettent de lutter contre la transmission du SRAS-CoV-2 [ou SARS si vous préférez]. Il s’agit d’un parcours de formation comprenant un serious game ludique et informatif illustré par Eric Buche. Ce parcours a été créé par une équipe transdépartementale des Hôpitaux Universitaires de Genève et intégrant des spécialistes en prévention et contrôle de l'infection

Nous vous recommandons d'encourager fortement vos collaborateurs, quel que soit leur fonction (soignants comme non-soignants), à effectuer ce parcours. Les données récoltées seront traitées de manière parfaitement anonyme, et vos collaborateurs n'auront qu'une donnée personnelle à saisir: leur adresse e-mail (personnelle ou professionnelle). Leurs noms et prénoms ne seront jamais demandés, et les adresses e-mails seront stockées dans une base de données cryptée située sur sol helvétique. Elles ne seront jamais révélées à des tiers et vos collaborateurs resteront parfaitement anonymes. Les données récoltées ne seront employées qu'à des fins de recherche contre la pandémie COVID-19.

A la fin du parcours, et après avoir vu le serious game et accédé aux normes Vigigerme des HUG, vos collaborateurs recevront un certificat de formation (au format PDF) qu'ils pourront sauvegarder ou imprimer.

Voici les informations de connection:
1. Se connecter à l'adresse internet: https://covid-escape.anesth.ch
2. Choisir son institution d'appartenance
3. Cliquer sur "Accéder"
4. Renseigner les champs - l'accréditation de votre institution est: ###

Le parcours dure environ 30 minutes.

Pour toute question, vous pouvez contacter seriousgame.covid19@hcuge.ch
